# Optimization of Unnicked ****β****2-Glycoprotein I and High Avidity Anti-****β****2-Glycoprotein I Antibodies Isolation

**DOI:** 10.1155/2014/195687

**Published:** 2014-01-23

**Authors:** Andrej Artenjak, Adrijana Leonardi, Igor Križaj, Aleš Ambrožič, Snezna Sodin-Semrl, Borut Božič, Saša Čučnik

**Affiliations:** ^1^Department of Rheumatology, University Medical Centre Ljubljana, Laboratory for Immunology, Vodnikova 62, 1000 Ljubljana, Slovenia; ^2^Department of Molecular and Biomedical Sciences, Jožef Stefan Institute, 1000 Ljubljana, Slovenia; ^3^Department of Chemistry and Biochemistry, Faculty of Chemistry and Chemical Technology, University of Ljubljana, 1000 Ljubljana, Slovenia; ^4^Centre of Excellence for Integrated Approaches in Chemistry and Biology of Proteins, 1000 Ljubljana, Slovenia; ^5^Natural Sciences and Information Technologies, Faculty of Mathematics, University of Primorska, 6000 Koper, Slovenia; ^6^Faculty of Pharmacy, University of Ljubljana, 1000 Ljubljana, Slovenia

## Abstract

Patient biological material for isolation of **β**2-glycoprotein I (**β**2GPI) and high avidity IgG anti-**β**2-glycoprotein I antibodies (HAv anti-**β**2GPI) dictates its full utilization. The aim of our study was to evaluate/improve procedures for isolation of unnicked **β**2GPI and HAv a**β**2GPI to gain unmodified proteins in higher yields/purity. Isolation of **β**2GPI from plasma was a stepwise procedure combining nonspecific and specific methods. For isolation of polyclonal HAv a**β**2GPI affinity chromatographies with immobilized protein G and human **β**2GPI were used. The unknown protein found during isolation was identified by liquid chromatography electrospray ionization mass spectrometry and the nonredundant National Center for Biotechnology Information database. The average mass of the isolated unnicked purified **β**2GPI increased from 6.56 mg to 9.94 mg. In the optimized isolation procedure the high molecular weight protein (proteoglycan 4) was successfully separated from **β**2GPI in the 1st peaks with size exclusion chromatography. The average efficiency of the isolation procedure for polyclonal HAv anti-**β**2GPI from different matrixes was 13.8%, as determined by our *in-house* anti-**β**2GPI ELISA. We modified the *in-house* isolation and purification procedures of unnicked **β**2GPI and HAv anti-**β**2GPI, improving the purity of antigen and antibodies as well as increasing the number of tests routinely performed with the *in-house* ELISA by ~50%.

## 1. Introduction

Recent findings of antiphospholipid syndrome (APS) pathogenesis support the important role of *β*2-glycoprotein I (*β*2GPI), as one of the most studied antigens [1–3]. *β*2GPI is a ~50 kDa protein with a mean plasma concentration in the healthy population of ~180 mg/L. The protein consists of 326 amino acids folded into 5 domains [4, 5]. The first 4 domains contain approximately 60 amino acids, whereas the last, 5th domain consists of 82 amino acids containing specific segments of positively charged amino acids ^281^CKNKEKKC^288^ and a hydrophobic loop ^313^LAFW^316^, which along with 19 amino acids of the C-terminal extension form the binding site to negatively charged phospholipids [6, 7]. Plasmin can clip/nick *β*2GPI at amino acids L^317^T^318^ and consequently terminate its ability to bind phospholipids [8]. Furthermore, recently it was observed that *β*2GPI can exist in different conformations, that is, in a circular form that can change to an open (fishhook) conformation after exposure to anionic structures or negatively charged phospholipids, which can be stabilized by anti-*β*2GPI antibodies (anti-*β*2GPI) [9, 10].

The presence of anti-*β*2GPI in human sera or plasma is one of the defining laboratory criteria for classification of APS [3, 11]. High avidity IgG anti-*β*2GPI (HAv anti-*β*2GPI) represent a subgroup of anti-*β*2GPI associated with thrombotic [12, 13] and obstetric [14] manifestations in APS patients. On the other hand, several studies in past decades implied that anti-*β*2GPI were associated with the development of atherosclerosis in autoimmune patients (as reviewed in [15]) and represent a non-traditional risk factor for atherosclerosis-based cardiovascular diseases in patients without overt autoimmunity (reviewed in [16]).

In the context of fully utilizing the preparatory fractions for the isolation of human unnicked *β*2GPI and HAv anti-*β*2GPI, as well as optimizing their yield, we evaluated and improved the protocols and procedures/methods involved [17, 18]. The primary aim was to gain unmodified, endogenous proteins in higher yields and purity.

## 2. Materials and Methods

### 2.1. Isolation of *β*2-Glycoprotein I

For isolation of human unnicked *β*2GPI, AB plasma pooled from apparently healthy donors was used. Isolation included a stepwise procedure combining perchloric acid (PA) precipitation, heparin affinity, and cationic exchange chromatography as determined by Cucnik et al. [17], with modifications performed in the precipitation step. In contrast to the previously used isolation procedure (standard protocol) [17], precipitation with PA was carried out in 3 aliquots of 80–90 mL of plasma starting volume (~250 mL) diluted with an equal volume of physiological solution of sodium chloride. Precipitation was carried out in an ice bath (0°C) using 60% PA, which was added dropwise to a final concentration of 0.285 M. The addition of PA in the optimized protocol lasted ~20 min per aliquot, as compared to the standard protocol which involved a precipitation step lasting ~60 min or more, due to the higher starting volume of plasma (~250 mL). Immediately after precipitation the suspension was centrifuged at 4°C and the pH of supernatants was adjusted to 8.0 with 1 M NaOH. Following precipitation, centrifugation and adjustment of pH, all aliquots were combined and dialyzed against 0.02 M Tris-HCl/0.03 M NaCl, pH 8.0 overnight. The following steps were without modifications as previously described [17]. Briefly, the dialysed supernatants were concentrated using 350 mL Amicon Stirred Ultrafiltration cell unit (Millipore, Bedford, MA, USA) and Ultrafiltration Membranes from regenerated cellulose with a molecular weight cut-off (MWCO) lower than 10 kDa (Millipore, Carrigtwohill Co., Cork, Ireland). In the next step concentrated dialysed supernatants were applied to a 10 mm × 20 cm Heparin Sepharose CL-6B column (GE Healthcare Bio-Sciences AB, Uppsala, Sweden), which was equilibrated with the same buffer. For elution of bound proteins 0.02 M Tris-HCl/0.35 M NaCl, pH 8.0 was used. Eluted fractions containing proteins were then pooled and dialysed against 0.05 M acetate buffer/0.05 M NaCl, pH 4.8 overnight and finally applied to a 5 × 50 mm cation exchange column with polystyrene/divinylbenzene matrix and R-CH_2_-SO^3−^ charged groups (Mono S 5/50 GL, GE Healthcare Bio-Sciences AB, Uppsala, Sweden). *β*2GPI was eluted over a linear gradient starting from 0.05 M NaCl at pH 4.8 to 0.65 M NaCl at pH 5.2 (Na^+^ in acetate buffer). After the last step (cationic exchange chromatography), three quantitatively different peaks were collected and dialyzed against phosphate buffered saline, pH 7.4 (PBS) with 0.02% NaN_3_ for 2 h (repeated twice). Protein concentrations were determined by NanoDrop 2000c Spectrophotometer (Thermo Fischer Scientific, Wilmington, Delaware, USA) at a wavelength of 280 nm using the excitation coefficient for *β*2GPI *E*_1 cm_^1%^ = 10.0. The final preparations were aliquoted and stored at −80°C for further analysis and/or use.

### 2.2. Polyacrylamide Gel Electrophoresis

The purity of isolated *β*2GPI was checked by sodium dodecyl sulphate-polyacrylamide gel electrophoresis (SDS-PAGE) according to Laemmli protocol [19] in 4% stacking and 10% resolving gels on the Mini-Protean II apparatus (Bio-Rad Laboratories, Hercules, CA, USA). Briefly, isolated protein samples were diluted in SDS sample buffer with 2-mercaptoethanol as reduction agent. Samples were then heated at 98°C for 5 min and afterwards applied to the gels. Electrophoresis was run at 125 V (stacking gel) and 250 V (resolving gel) at 4°C. Staining was carried out with Coomassie Brilliant Blue R250 (CBB) and destaining with a 10% acetic acid solution in 25% ethanol. Gels were scanned using G-Box (Syngene, Cambridge, United Kingdom).

### 2.3. Mass Spectrometry Analysis

Liquid chromatography electrospray ionization mass spectrometry (LC-ESI-MS/MS) was used to determine an unknown protein band ~150–250 kDa coming from the 1st peaks of column chromatography and detected on 10% SDS-PAGE. Due to the higher purity demands for ESI-MS/MS analysis (to avoid keratin and any other contamination), samples were run on a premade 7.5% polyacrylamide gel (Bio-Rad Laboratories, Hercules, CA, USA) at 250 V in a laminar air flow chamber. All samples and buffers were filter sterilized. After staining with CBB and destaining with 30% methanol, the stained band was cut out and stored at −20°C in sterile autoclaved microtubes for further MS analysis. Further destaining was done with 25 mM ammonium bicarbonate/50% (V/V) acetonitrile and in-gel digestion was performed using mass spectrometry grade modified trypsin (Promega, Madison, WI, USA) in 25 mM ammonium bicarbonate overnight at 37°C. The resulting peptides were extracted with 50% acetonitrile/5% formic acid (V/V), concentrated and analyzed on an ion trap mass spectrometer 1200 series HPLC-Chip-LC/MSD Trap XCT Ultra (Agilent Technologies, Waldbronn, Germany). MS and MS/MS spectra were searched against the non-redundant National Center for Biotechnology Information (NCBI-nr) database using the Mascot software (Matrix Science Ltd., UK).

### 2.4. Size Exclusion Chromatography

Size exclusion chromatography was carried out using Sephacryl S-300 HR, as matrix on a 16 mm × 100 cm column (16/100 XK, Pharmacia Biotech, Uppsala, Sweden) in order to separate *β*2GPI from proteoglycan 4 (PRG4) in the 1st peaks with a fractionation range for globular proteins from 10 to 1500 kDa [20]. The 1st peaks from several *β*2GPI isolations were collected and concentrated with Amicon Ultra centrifugation filters from regenerated cellulose with MWCO lower than 10 kDa (Millipore, Carrigtwohill Co., Cork, Ireland) at 2000 ×g. The final volume of ~2.5 mL was applied to the column with PBS as the mobile phase. The flow used was 10 mL/h and the volume of collected fractions was ~2.3 mL. Fractions from corresponding peaks were concentrated using Amicon Ultra centrifugation filters from regenerated cellulose with MWCO 30 kDa (Millipore Corporation, Billerica, MA, USA) and stored at −20°C.

### 2.5. *In-House* Anti-*β*2-Glycoprotein I ELISA

*In-house* anti-*β*2GPI ELISA was used to check the functionality of purified unnicked *β*2GPI [21, 22]. The test was conducted with seven sera samples from different autoimmune patients, positive and negative controls with respect to IgG, IgM, and IgA subtypes. Results were presented in arbitrary IgG, IgM, and IgA titer units (negative < 2, positive ≥ 2 and high positive ≥ 16).

Avidity of patient samples used for isolation of anti-*β*2GPI was determined using a chaotropic variant of the *in-house* anti-*β*2GPI ELISA. Anti-*β*2GPI were arbitrarily defined as HAv when the binding in PBS-0.05% Tween-20 (PBS-Tw) with 0.5 M NaCl remained 65% or higher than the initial binding in PBS-Tw (0.15 M NaCl) [18, 23].

### 2.6. Isolation of High Avidity Anti-*β*2-Glycoprotein I Antibodies

For isolation of polyclonal HAv anti-*β*2GPI from human sera, plasma, or immunoadsorption fractions, a stepwise procedure combining affinity chromatographies with immobilized protein G and human unnicked *β*2GPI were used [18]. Improvements of the elution step from the *β*2GPI affinity column were done as previously described [19, 24].

IgG fractions were concentrated to volumes of <50 mL using Ultrafiltration Membranes from regenerated cellulose with MWCO 50 kDa (Millipore Corporation, Billerica, MA, USA) in a 350 mL Amicon Stirred Ultrafiltration Cell unit (Millipore, Bedford, MA, USA) at 4°C and applied onto a column with immobilized human unnicked *β*2GPI (30 mg of pure unnicked *β*2GPI was coupled with 10 mL of CNBr-activated Sepharose 4B (Sigma-Aldrich, St Louis, MO, USA)) [18]. HAv anti-*β*2GPI were eluted from the column with 0.1 M glycine/4 M NaCl, pH 2.5 followed by PBS. These two steps were repeated 3 times. Fractions were immediately neutralized with neutralization buffer to pH ~7 and then dialyzed against PBS at 4°C overnight. Afterwards, HAv anti-*β*2GPI were concentrated as described above, using Amicon Ultra centrifugation filters from regenerated cellulose with MWCO 30 kDa, followed by sterile filtration (0.2 *μ*m; Minisart, Sartorius Stedim Biotech GmbH, Goettingen, Germany). The concentrations of HAv anti-*β*2GPI in each step of isolation were derived from the standard curve according to the defined dilutions of IgG Sapporo Standard (HCAL; INOVA Diagnostics, San Diego, CA, USA) by *in-house* anti-*β*2GPI ELISA [21, 22].

### 2.7. Statistical Analysis and Data Presentation

Data are presented in mean values ± standard deviation (SD). Where statistical analysis was applied, each set of data was normally distributed according to Shapiro-Wilkes test. For statistical analysis the two-tail Student's *t*-test with significance level *P* < 0.05 was used.

## 3. Results

**Figure 1:**
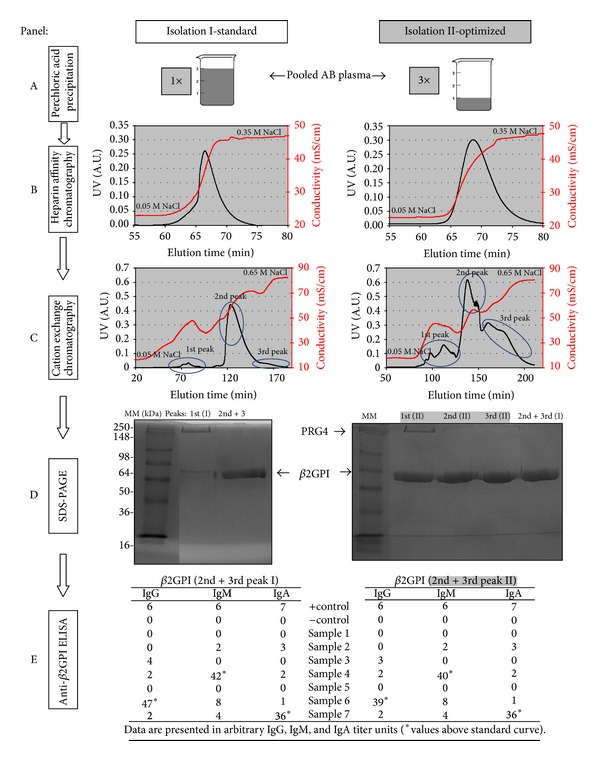
Flow chart and graphical data of**** *β*2-glycoprotein I isolation procedure: comparison between isolation data after standard (isolation I) and optimized (isolation II) protocols. Panel A: the perchloric acid precipitation was carried out with the whole volume (V) (isolation I) and in 3 aliquots (isolation II) from the same starting material (pooled plasma from 2 AB donors, see Table 1). Panel B: elution chromatograms with NaCl gradient after heparin affinity chromatography. Panel C: elution chromatograms with NaCl gradient after cation exchange chromatography. Panel D: purity check of protein fractions collected after cationic exchange chromatography as detected by Coomassie Brilliant Blue stained 10% SDS-PAGE (~5 *μ*g of proteins/lane). Indicated are *β*2GPI in 2nd and 3rd peaks of isolations I and II, respectively, and PRG4 in 1st peak after optimized protocol (isolation II). Panel E: functionality check of isolated *β*2GPI. Pure *β*2GPI was used as antigen in anti-*β*2GPI ELISA. Data is presented in arbitrary IgG, IgM, and IgA units—negative < 2, positive ≥ 2, and high positive ≥ 16. Legend: A.U.: absorbance units; *β*2GPI: *β*2-glycoprotein I; MM: molecular weight marker; mS/cm: millisiemens per centimeter; PRG4: proteoglycan 4; SDS-PAGE: sodium dodecyl sulphate-polyacrylamide gel electrophoresis.

**Scheme 1:**
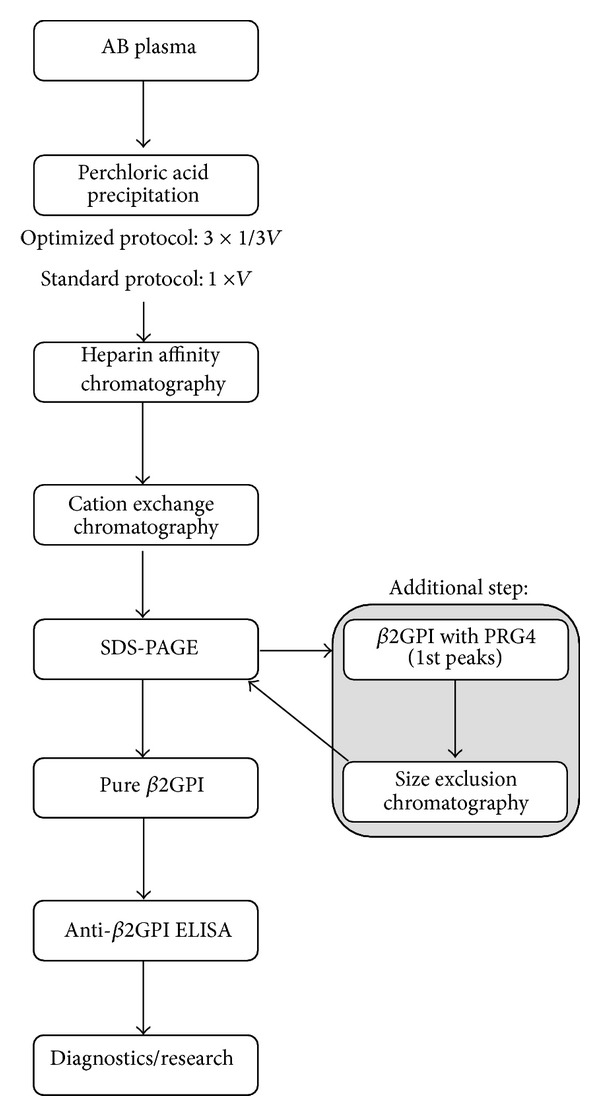
*β*2-Glycoprotein I isolation procedure after optimization and using size exclusion chromatography as an additional step. *β*2GPI: *β*2-glycoprotein I; PRG4: proteoglycan 4.

Modification of the *β*2GPI isolation procedure with special emphasis on duration of precipitation with PA improved the efficiency of the procedure by 51.5% (Table 1, Scheme 1). The precipitation was carried out in three smaller aliquots (~80–90 mL) from similar starting volumes (~250 mL), regardless of the isolation procedure. Lowering the precipitation volume (i.e., dividing the starting volume to smaller aliquots which were precipitated separately) consequently shortened the duration of PA precipitation from an average of 63 ± 6 min to 20 ± 1 min per aliquot. Elution from cationic exchange chromatography resulted in three protein peaks (Figure 1). After purity and functionality check with SDS-PAGE and anti-*β*2GPI ELISA, the isolated mass of unnicked human *β*2GPI rose significantly from 6.56 ± 1.38 mg (*n* = 12; range 3.63 to 8.85 mg) using the standard protocol of isolation [17] to 9.94 ± 1.57 mg (*n* = 5; range 8.69 to 12.56 mg) (*P* = 0.004), following optimization of the standard protocol.

In one example, isolation data were compared after *β*2GPI isolation according to standard (isolation I) and optimized (isolation II) protocols from the same starting material, pooled from two AB plasma donors (Table 1, Figure 1). The starting volumes were 250 mL and 255 mL, with the precipitation time ~61 min according to standard and ~21 min per aliquot according to the optimized protocol, respectively. After elution from heparin affinity column the peak was higher in isolation II as compared to isolation I (Figure 1, Panel B). The next step yielded 3 peaks in isolations I and II (Figure 1, Panel C). Since in isolation I the total protein mass of the 3rd peak was very low and the amount of protein C inhibitor was negligible [17], this peak was not further separately examined. The purity check with 10% SDS-PAGE revealed 3 peaks with different quantity of *β*2GPI in isolation II, which were not different as compared to the band of unnicked *β*2GPI from the 2nd and 3rd peaks after the standard procedure (Figure 1, Panel D, bands >50 kDa). In the 1st peaks of both isolations a protein band with molecular weight over 250 kDa was present (described below). The functionality check with anti-*β*2GPI ELISA showed no differences between pure *β*2GPI isolated after the new optimized protocol (combined 2nd and 3rd peaks—isolation II) as compared to the unnicked *β*2GPI isolated according to the standard protocol (2nd peak—isolation I) (Figure 1, Panel E). In this specific case, the mass of isolated unnicked *β*2GPI rose by more than 41% (from 7.22 mg to 10.21 mg) (Table 1).

In the optimized isolation procedure we observed an impurity of high molecular weight (150–250 kDa) in the 1st peak (Figure 1, Panel D, isolation II). The unknown protein was determined to be proteoglycan 4 (PRG4; megakaryocyte stimulating factor or lubricin) using LC-ESI-MS/MS and NCBI-nr database searching of tryptic peptides. PRG4 was an impurity present in the highest amounts in the 1st peak. It was successfully separated from unnicked *β*2GPI by size exclusion chromatography.

From a functional prospective, 50 *μ*L of 1 g/L *β*2GPI is routinely used for 96-well plates in our *in-house* ELISA for anti-*β*2GPI determination, which is sufficient for carrying out 48 tests (technical duplicates). Through the improvement of the isolation procedure, the calculated estimate for diagnostic use increased from ~6300 to ~9500 tests, gaining an additional ~3200 tests.

The average efficiency of the isolation procedure for polyclonal IgG anti-*β*2GPI from plasma on the protein G and *β*2GPI column was 8.9%, as determined by our *in-house* anti-*β*2GPI ELISA (Table 2). The average efficiency from different starting materials, such as immunoadsorption, sera, and plasma was 13.8% (ranging from 6% to 21.4%; data not shown). All isolated samples were established as HAv anti-*β*2GPI, due to their binding to antigen in PBS-Tw in a higher ionic strength environment (0.5 M NaCl) being ≥80% of the initial binding in PBS-Tw (0.15 M NaCl).

## 4. Discussion

*β*2GPI and anti-*β*2GPI are important proteins in the pathology of APS, especially since anti-*β*2GPI represent one of the diagnostic markers/laboratory criteria for the disease classification. The main pathological functions of anti-*β*2GPI in APS rise from complexes formed by anti-*β*2GPI binding to *β*2GPI in the fishhook-open conformation and impacting the vascular system [25].

In order to improve the existing procedure for isolation of unnicked *β*2GPI, we found that the time of protein exposure to low pH was important. According to recent findings by Ağar et al. [9], we assume that the isolated and solubilized unnicked form of *β*2GPI is present in circular conformation, whereas in the anti-*β*2GPI ELISA *β*2GPI is expected to be unfolded, due to the physical and chemical characteristics of the microtiter plates. This would allow for hydrophobic and ionic binding (with respect to negatively charged carboxyl groups) to the fishhook conformation of *β*2GPI, making detection of anti-*β*2GPI possible and reproducible [21, 22]. Brighton et al. reported that purification of *β*2GPI using PA precipitation yields from 49.7–90.8% of unnicked *β*2GPI, whereas 9.2–50.3% of purified *β*2GPI was reported to be in the cleft form, depending on the elution peaks collected in the last step. Observed cleavage occurred at Lys^317^Thr^318^ and to a lesser extent at Ala^314^Phe^315^ as determined by N-terminal sequencing. Furthermore, in ELISA binding assays they observed no detectable binding to solid-phase anionic phospholipids in samples where 50% of *β*2GPI was cleft (~50% of *β*2GPI was unnicked), emphasizing the necessity of preserved structural integrity of *β*2GPI. The group speculated that *β*2GPI can be damaged in the above-mentioned segments in this isolation procedure, due to strong oxidative properties of PA [26]. Contrary to their observations, Cucnik et al. reported that the isolation with PA precipitation under controlled conditions, however different to Brighton et al. [26], yields unnicked *β*2GPI (wild-type) in each elution peak, as determined by SDS-PAGE, N-terminal sequencing, and anti-*β*2GPI ELISA [17]. If present, nicked *β*2GPI, could be found in quantities of <5%. One of the explanations for the different yields of unnicked/nicked *β*2GPI between the two reports could be the temperature at which precipitation with PA is carried out. Specifically, according to the standard protocol [17] Cucnik et al. added PA dropwise either for 3, 18, or 50 min on an ice bath (0–4°C), as compared to Brighton et al., where precipitation was conducted at room temperature (25°C) for ~15 min [26]. It could well be postulated that the lower temperature significantly slows the damaging and cleavage of *β*2GPI by PA. In the current report the time of precipitation with PA was significantly reduced to ~20 min, thus shortening the time of *β*2GPI contact with PA, as well as time of *β*2GPI in low pH. Compared to unnicked *β*2GPI gained after the standard protocol [17], the purified *β*2GPI after optimization exhibited similar characteristics on SDS-PAGE (Figure 1, Panel D) and anti-*β*2GPI ELISA (Figure 1, Panel E), indicating that after optimization we gain also structurally unmodified (e.g., unnicked) *β*2GPI.

Previously, the standard isolation procedure yielded three qualitatively different protein peaks with different quantity and purity of *β*2GPI [17]. In the 1st peaks also immune complexes of immunoglobulins and *β*2GPI were detected, the 2nd peaks yielded only *β*2GPI, whereas concomitant presence of *β*2GPI and protein C inhibitor was reported in the 3rd quantitatively smallest peaks [17]. Currently, we describe that using a modification of the *β*2GPI isolation procedure, the 1st elution peaks after cationic exchange chromatography, yielded a newly detected protein identified by LC-ESI-MS/MS as PRG4. This is a highly glycosylated human protein consisting of 1404 amino acids with a core molecular weight of ~150 kDA, ranging up to 400 kDa, depending on the O-glycosylation and glycosaminoglycan substitutions [27]. The main function of PRG4 is lubrication of articular cartilage in joints [28]. In synovial fluid, the observed average concentrations can be ~290 mg/L (287.1 ± 31.8 mg/L) [29], whereas in serum/plasma the concentrations of PRG4 have, to our knowledge, not been reported to date. For separation of *β*2GPI from PRG4 in the 1st peaks, a new step was added to the optimized protocol, specifically size exclusion chromatography (Scheme 1).

For diagnostic purposes of APS, the presence and quantity of anti-*β*2GPI are crucial. A subpopulation of anti-*β*2GPI, specifically HAv anti-*β*2GPI, was successfully isolated in the current report. The procedure involved multiple steps [24] including affinity binding onto a column with immobilized unnicked *β*2GPI. The average and the highest isolation efficiency from human sera, plasma, or immunoadsorption were determined by *in-house* anti-*β*2GPI ELISA as 13.8% and 21.4%, respectively. HAv anti-*β*2GPI present a clinically relevant subpopulation of anti-*β*2GPI, which can correlate with thrombosis in patients with APS [12, 13]. This correlation, as well as association of HAv anti-*β*2GPI with obstetric complications, was recently reported by Cucnik et al. [14, 30]. These data were confirmed by the European multicenter study analysing HAv anti-*β*2GPI that enrolled 226 out of 479 patients with primary APS and APS associated with other autoimmune diseases as well as patients with other non-APS autoimmune diseases [14]. Recently, it was also observed that HAv anti-*β*2GPI influenced human coronary artery endothelial cells to release chemotactic and inflammatory cytokines which consequently resulted in a higher migration of peripheral blood mononuclear cells to preconditioned supernatants [24].

## 5. Conclusions

Modification of the *in-house* isolation and purification procedures for unnicked *β*2GPI and polyclonal IgG anti-*β*2GPI of high avidity led to increased purity of both as well as a substantial elevation in the number of diagnostic tests performed.

## Figures and Tables

**Table 1:** *β*2-Glycoprotein I antigen isolation procedure: comparison of standard and optimized protocols.

	Standard protocol	Optimized protocol	*β*2GPI isolation from the same starting material after both protocols	
AB plasma donors (*n*)	14	6	2	
Number of isolations	12	5	1 (isolation I)	1 (isolation II)
Starting volume, mean ± SD (mL)	254 ± 24	246 ± 8	250	255
Number of aliquots	1	3	1	3
Precipitation duration, mean ± SD (min)	63 ± 6	20 ± 1	61.25	21.25
Mass of isolated *β*2GPI, mean ± SD (mg)	6.56 ± 1.38*	9.94 ± 1.57*	7.22	10.21
Efficiency improvement	51.5%		41.4%	

*β*2GPI, *β*2-glycoprotein I. *Significantly higher mass (*P* = 0.004) as compared to standard protocol (Student's *t*-test, two-tail).

**Table 2:** Isolation of human polyclonal high avidity IgG antibodies against *β*2-glycoprotein I: a representation of isolation efficiency calculation.

Starting material (plasma)	*V*_*o*_ (mL)	205
	*C_*o*_ HAv anti-*β*2GPI (*μ*g/mL)	33.9
	*m_*o*_ anti-*β*2GPI (*μ*g)	6950
1. step	*m_start_ HAv anti-*β*2GPI (*μ*g)	6950
IgG isolation	*m_isolated_ HAv anti-*β*2GPI (*μ*g)	6049
(*protein G column*)	*η* (1. step)	0.870
2. step	*m_start_ HAv anti-*β*2GPI (*μ*g)	6049
HAv a*β*2GPI isolation	*m_isolated_ HAv anti-*β*2GPI (*μ*g)	620
(*β2GPI column*)	*η* (2. step)	0.102
	*η* total (%)	8.9%

Concentrations and calculated masses were determined by *in-house* anti-*β*2GPI ELISA (*). *β*2GPI: *β*2-glycoprotein I; HAv anti-*β*2GPI: high avidity anti-*β*2GPI IgG antibodies; *V*_*o*_: volume of plasma used for isolation; *C*_*o*_: starting concentrations of HAv anti-*β*2GPI; *m*_*o*_, *m*_start_, and *m*_isolated_: calculated masses of HAv anti-*β*2GPI before isolation and before and after each step; and *η*: estimated efficiency of isolation.
